# Microcirculation and Macrocirculation in Cardiac Surgical Patients

**DOI:** 10.1155/2012/654381

**Published:** 2012-06-05

**Authors:** Elli-Sophia Tripodaki, Athanasios Tasoulis, Antigoni Koliopoulou, Ioannis Vasileiadis, Leonidas Vastardis, Giorgos Giannis, Mihalis Argiriou, Christos Charitos, Serafim Nanas

**Affiliations:** ^1^First Critical Care Department, Evangelismos Hospital, National and Kapodistrian University of Athens, Ypsilantou 45–47, 106 75 Athens, Greece; ^2^2nd Department of Cardiac Surgery, Evangelismos Hospital, 106 75 Athens, Greece

## Abstract

*Background*. The aim of our study was to investigate the relationship between microcirculatory alterations after open cardiac surgery, macrohemodynamics, and global indices of organ perfusion. *Methods*. Patients' microcirculation was assessed with near-infrared spectroscopy (NIRS) and the vascular occlusion technique (VOT). *Results*. 23 patients undergoing open cardiac surgery (11 male/12 female, median age 68 (range 28–82) years, EuroSCORE 6 (1–12)) were enrolled in the study. For pooled data, CI correlated with the tissue oxygen consumption rate as well as the reperfusion rate (*r* = 0.56, *P* < 0.001 and *r* = 0.58, *P* < 0.001, resp.). In addition, both total oxygen delivery (DO_2_, mL/min per m^2^) and total oxygen consumption (VO_2_, mL/min per m^2^) also correlated with the tissue oxygen consumption rate and the reperfusion rate. The tissue oxygen saturation of the thenar postoperatively correlated with the peak lactate levels during the six hour monitoring period (*r* = 0.50, *P* < 0.05). The tissue oxygen consumption rate (%/min) and the reperfusion rate (%/min), as derived from the VOT, were higher in survivors compared to nonsurvivors for pooled data [23 (4–54) versus 20 (8–38) *P* < 0.05] and [424 (27–1215) versus 197 (57–632) *P* < 0.01], respectively. *Conclusion*. Microcirculatory alterations after open cardiac surgery are related to macrohemodynamics and global indices of organ perfusion.

## 1. Introduction

Cardiac surgery is characterized by microcirculatory alterations and reduced organ perfusion, due to a combination of the surgery itself, the anesthesia, the hypothermia, the hemodilution, the microemboli formation that occur during the procedure [1, 2], and mainly the intense systematic inflammatory response that develops and peaks the first twenty four hours postoperatively [3]. Peripheral blood flow and oxygen supply can also be affected postoperatively by a low cardiac output state. This can occur in patients with compromised systolic or diastolic ventricular function but also as a result of myocardial “stunning” due to ischemia-reperfusion injury of the heart. If left without intervention, it can also lead to tissue damage and organ failure [4]. It has been observed that microcirculatory derangements may be present despite systemic hemodynamics being within satisfactory goals [5, 6].

Near-infrared spectroscopy (NIRS) is a noninvasive, bed side easily applicable tool that has been used to provide an estimate of tissue oxygenation in health and in disease [7, 8]. By performing a vascular occlusion technique (VOT) NIRS can be used at rest [9, 10] and during interventions [11, 12] for the evaluation of the microcirculation.

We hypothesized that the microcirculatory alterations after cardiac surgery as assessed by NIRS technology are related to macrocirculatory indices.

The aim of our study was to investigate a possible relationship between NIRS derived parameters and macrohemodynamics, as well as global indices of organ perfusion, in cardiac surgical patients.

## 2. Materials and Methods

### 2.1. Study Design

We conducted an observational study at Evangelismos hospital, a 1000-bed tertiary hospital. Patients undergoing planned cardiac surgery with cardiopulmonary bypass were included in the study. The study was approved by the Scientific Council and the Ethics Committee of our Hospital and informed consent was obtained from all patients. Before the operation, in-hospital mortality risk was predicted with the EuroSCORE [13]. Data collection included near-infrared spectroscopy (NIRS) measurements upon routine admission to the cardiac Intensive Care Unit (cICU) postoperatively and every two hours for a six hour monitoring period as well as hemodynamic measurements.

### 2.2. Anesthesia, Surgery, and CBP Management

Before induction of anesthesia, an arterial line was placed into the radial artery. Anesthesia was intravenously induced with midazolam, fentanyl, hypnomidate or propofol, and cis-atracurium, and it was maintained with sevoflurane, or propofol, fentanyl and cis-atracurium. After tracheal intubation, the lungs were volume controlled with a tidal volume of 8–10 mL/kg resulting in an end-tidal CO_2_ concentration between 4 and 5%, using an O_2_-air mixture with an inspiratory O_2_ concentration of 40%. A positive-end expiratory pressure (PEEP) of 5 cm H_2_O was applied. Nonpulsatile CPB was established through a standard median sternotomy with aortic root and right heart cannulation. Surgery was performed under temperatures ranging between 30 and 32°C. Anticoagulation was established with intravenous heparin (3 mg/kg) given 10 minutes before initiation of CPB with target-activated clotting time being at least 440 seconds. After aortic cross clamping, 1000 cc of cold blood cardioplegia were administered, and this was repeated every 20 min thereafter. At the end of CPB, the patient was rewarmed, and heparin was reversed with intravenous protamine sulphate (4 mg/kg). After the operation, patients were admitted to the cICU where they remained until they were hemodynamically stabile and extubated, when they returned to the ward.

### 2.3. Macrohemodynamic Monitoring

In the cICU cardiac output (CO) was monitored with the thermodilution technique with the insertion of a pulmonary artery catheter (19 patients) or with the pulse contour analysis method (4 patients). Arterial and central venous pressure was monitored invasively in all cases. During the postoperative period, patients were resuscitated according to the following parameters [4]: mean arterial pressure (MAP) ≥ 60 mmHg, central venous pressure between 8 and 12 mmHg, cardiac index (CI) ≥ 2.2 L/min/m^2^, hemoglobin concentration (Hb) between 8 and 10 g/dL, SvO_2 _≥ 65%, SaO_2 _≥ 96%, blood glucose ≤ 160 mg/dL.

Total oxygen delivery (DO_2_), total oxygen consumption (VO_2_), and the oxygen extraction ratio were calculated for every patient at each measurement.

### 2.4. Microcirculatory Assessment and Analysis

Near-infrared spectroscopy is a noninvasive method for continuous monitoring of tissue oxygenation. Although visible light is unable to penetrate biological tissue for more than 1 cm because it is strongly absorbed and scattered by tissue constituents (mainly water), light in the near-infrared region can easily reach much deeper biological structures. In mammalian tissue, only three compounds change their spectra when oxygenated: hemoglobin, myoglobin, and cytochrome aa3 [14]. As the absorption spectra of oxyhemoglobin and deoxyhemoglobin differ, a modified Beer-Lambert's law can be used to detect their relative concentrations within tissues. StO_2_ reflects the ratio of oxygenated hemoglobin to total hemoglobin. Because NIRS measurements are performed regardless of the diastolic or systolic phase and as only 20% of blood volume within tissue microcirculation is intra-arterial, spectroscopic measurements are primarily indicative of the venous oxyhemoglobin concentration. In our study, thenar tissue oxygen saturation (StO_2_) was measured using wide-gap second derivative NIRS (InSpectra; Hutchinson Technology). This technology provides an estimate of the hemoglobin saturation (StO_2_) in the microvasculature of muscle tissue, comprising the arteriolar, capillary, and venular compartments, according to principles described previously [15–19].

The measurements at each time point were made while a vascular occlusion technique was applied. After an initial resting StO_2_ value had been recorded on the thenar, a pneumatic cuff placed above the elbow was rapidly inflated to 50 mmHg above the patient's systolic arterial blood pressure and maintained for 3 minutes, after which it was released. Signal acquisition proceeded during the occlusion period and until StO_2_ values were again stabilized following cuff release. The vascular occlusion derived curves were stored using InSpectra software. StO_2_ curves were analyzed offline blindly and in random order (InSpectra Analysis Program, version 2.0; Hutchinson Technology; Hutchinson, MN; running in MatLab 7.0; The MathWorks; Novi, MI). The first degree slope of the hemoglobin desaturation curve during stagnant limb ischemia reflects the tissue oxygen consumption rate (%/min), and the slope of the increase of StO_2_ after the release of the brachial vascular occlusion is indicative of the reperfusion rate (%/min) [20–22].

The first NIRS measurement was performed upon cICU admission (H_0_), and then at two (H_2_), four (H_4_), and six hours (H_6_) postoperatively. At each time point, arterial and venous blood samples were drawn for the measurement of arterial and venous blood gases, lactate concentration, and ScvO_2_.

## 3. Statistical Analysis

All continuous variables are presented as median (range) or mean ± standard deviation. Analysis of variance (repeated measures ANOVA) and subsequent Bonferroni test were used to establish differences in microcirculatory parameters and global hemodynamic variables between the successive measurement periods. Pearson bivariate correlation was used to study the correlation of various parameters of the microcirculation with hemodynamic indices. The level of significance was set at <0.05.

## 4. Results

### 4.1. Study Population

Twenty three patients undergoing open cardiac surgery (11 male/12 female) of a median age of 68 (range 28–82) years were enrolled in the study. The patients had a median EuroSCORE of 6 (range 1–12). The surgical procedure performed is seen in Table 1. Patients were observed for 40 days postoperatively and 5 died within this period (time until death: median 14 days, range 5–39).

Upon routine admission to the cICU postoperatively, patients' circulation was supported with noradrenaline (1 patient), dobutamine (4), both (15), and levosimendan (1). One patient was supported with noradrenaline, adrenaline and levosimendan and one patient was not on inotropes/vasopressors upon cICU admission. All patients were sedated and mechanically ventilated throughout the six hour monitoring period.

The microcirculatory indices and the trend of the macrohemodynamics during the six-hour monitoring period postoperatively are presented in Tables 2(a) and 2(b).

### 4.2. Relationship between Microcirculation Parameters and Macrohemodynamics

**Figure 1:**
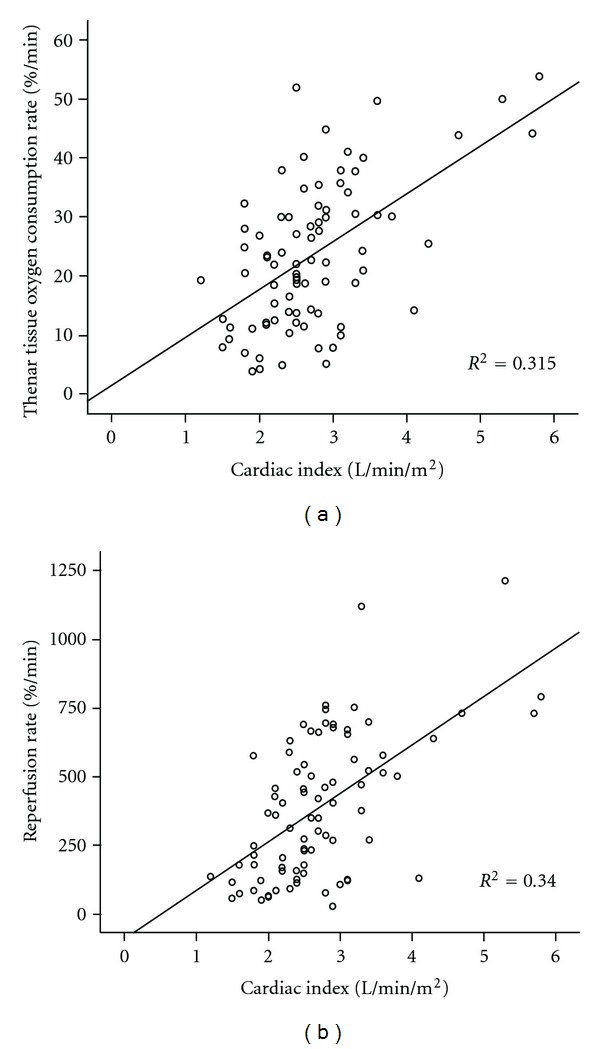
(a) Scattergram of the thenar tissue O_2_ consumption rate (%/min) during a 3 min vascular occlusion technique and cardiac index (L/min/m^2^, *r* = 0.56, *P* < 0.001, *n* = 82) for pooled data. (b) Scattergram of the reperfusion rate (%/min), indicative of the endothelial function after cuff release following a 3 min vascular occlusion technique and cardiac index (L/min/m^2^, *r* = 0.58, *P* < 0.001, *n* = 82) for pooled data.

**Figure 2:**
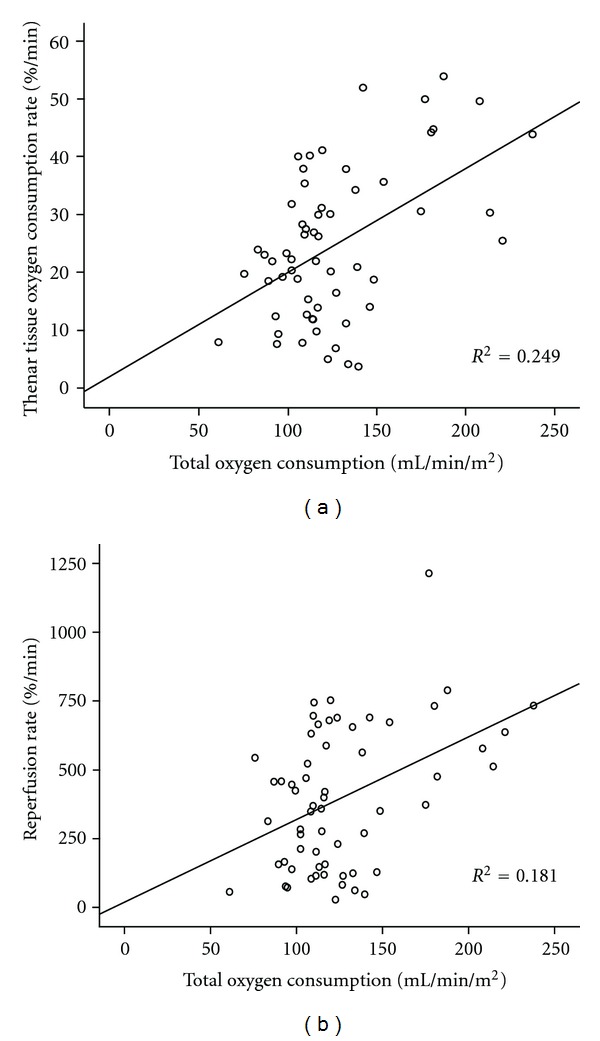
(a) Scattergram of the thenar tissue O_2_ consumption rate (%/min) during a 3 min vascular occlusion technique and total oxygen consumption (mL/min/m^2^, *r* = 0.50, *P* < 0.001, *n* = 60) for pooled data. (b) Scattergram of the reperfusion rate (%/min), indicative of, not or the endothelial function after cuff release following a 3 min vascular occlusion technique and total oxygen consumption (mL/min/m^2^) (*r* = 0.43, *P* < 0.001, *n* = 60) for pooled data.

For pooled data, a statistically significant correlation was found between cardiac index (CI) (L/min/m^2^) and microcirculatory parameters obtained by performing the vascular occlusion technique. Specifically, CI correlated with the tissue O_2_ consumption rate (%/min) as well as the reperfusion rate (%/min) (*r* = 0.56, *P* < 0.001 and *r* = 0.58, *P* < 0.001 resp., Figures 1(a) and 1(b)). This relationship remained significant when controlled for patients' temperature. In addition, both total oxygen delivery (DO_2_, mL/min per m^2^) and total oxygen consumption (VO_2_, mL/min per m^2^) also correlated with NIRS-derived parameters and specifically with the tissue O_2_ consumption rate and the reperfusion rate (*r* = 0.42, *P* < 0.001, and *r* = 0.43, *P* < 0.001 for DO_2_ and the tissue O_2_ consumption rate and the reperfusion rate, resp.) and (*r* = 0.50, *P* < 0.001 and *r* = 0.43, *P* < 0.001 for VO_2_ and the tissue O_2_ consumption rate and the reperfusion rate resp., Figures 2(a) and 2(b)). This relationship also remained significant when controlled for patients' temperature.

### 4.3. Relationship between Microcirculation Parameters and Global Indices of Organ Perfusion

When comparing VOT-derived microcirculatory indices amongst measurements with lactate levels up to 4 mg/dL (including 4) and lactate levels greater than 4, the first group had higher values of tissue O_2_ consumption rate and reperfusion rate (26 ± 11 versus 19 ± 13, *P* < 0.05 and  459 ± 237  versus 259 ± 240, *P* < 0.001, resp.).

The first measurement of the tissue oxygen saturation of the thenar (StO_2_ (%)) immediately after cICU admission (H_0_) postoperatively correlated with the peak lactate levels during the six hour monitoring period (*r* = 0.50, *P* < 0.05).

It is interesting to note the strong correlation between the tissue O_2_ consumption rate and the reperfusion rate (*r* = 0.79, *P* < 0.001).

**Figure 3:**
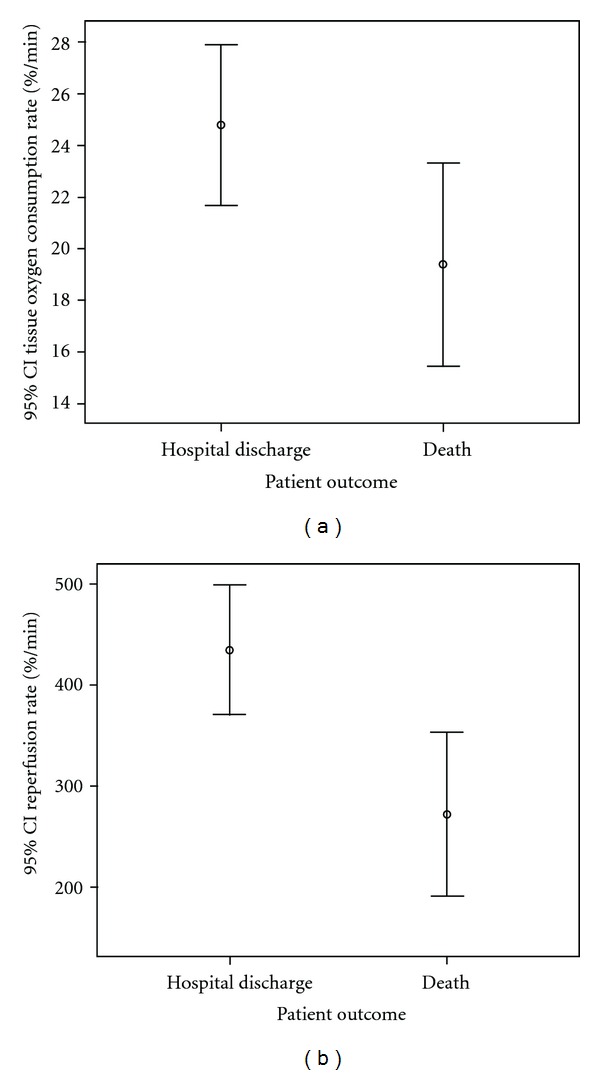
(a) Error bar of the thenar tissue O_2_ consumption rate (%/min) during a 3 min vascular occlusion technique in patient' measurements who survived to hospital discharge and patients who died, for pooled data (*P* < 0.05). (b) Error bar of the reperfusion rate (%/min) during a 3 min vascular occlusion technique in patient' measurements who survived to hospital discharge and patients who died, for pooled data (*P* < 0.01).

The tissue oxygen consumption rate was higher in survivors to hospital discharge compared to nonsurvivors (median 23 (range 4–54) versus 20 (8–38) *P* < 0.05), for pooled data (Figure 3(a)). Similarily, the reperfusion rate was higher in survivors compared to nonsurvivors (424 (27–1215) versus 197 (57–632) *P* < 0.01), for pooled data (Figure 3(b)).

## 5. Discussion

### 5.1. Relationship between Microcirculation Parameters and Macrohemodynamics

In our study, a significant relationship was found between patients' cardiac index and thenar tissue oxygen consumption rate as well as reperfusion rate. This is the first study to our knowledge that correlates NIRS and VOT-derived microcirculatory indices with macrohemodynamics in cardiac surgery patients. In a population of critically ill patients with septic shock, Payen et al. found a significant relationship between cardiac output and the reperfusion slope [23]. This relation may simply show that systemic flow influences peripheral StO_2_. Increase in cardiac index leads to increased regional perfusion and the subsequent improvement of microcirculatory indices.

Tissue oxygenation, tissue oxygen consumption rate, and the reperfusion slope gradually increased during the six hour monitoring period. However, a statistically significant increase was not established for more global indices of the patients' circulatory and oxygenation status, such as the CI, SvO_2_, DO_2_, and VO_2_.

The relationship between DO_2_ and VO_2_ and the tissue oxygen consumption rate and reperfusion rate can be interpreted by the fact that what happens regionally (i.e., at the site of the thenar muscle) is indicative of what happens globally. This relationship is not absolute though, as DO_2_ and VO_2_ are dependent on multiple parameters.

### 5.2. Relationship between Microcirculation Parameters and Global Indices of Organ Perfusion

The vascular occlusion-derived microcirculatory parameters and specifically the tissue oxygen consumption rate and the reperfusion rate were lower in the group with higher lactate levels. The SIRS which develops postoperatively and leads to microcirculatory alterations may also lead to increased lactate levels. The severity of microvascular alterations, as assessed by orthogonal polarization spectral imaging, also correlated with peak lactate levels after cardiac surgery in a study by de Backer et al. [1]. In the previously mentioned study by Payen et al., the reperfusion slope correlated with lactate levels [23]. The relationship between the thenar StO_2_ (%) immediately after cICU admission (H_0_) postoperatively and the peak lactate levels during the six-hour monitoring period suggests that the microvascular alterations were associated with impaired cellular oxygenation. 

It is worth mentioning the strong correlation between the tissue oxygen consumption rate and the reperfusion rate. A similar finding was noted by Payen et al. in the previously mentioned study [23]. The greater the tissue oxygen consumption rate, the faster oxygen is consumed in a given time period, which in turn leads to greater muscle ischemia and subsequent increased release of vasodilating substances. After the cuff is released, this may then lead to a faster reperfusion rate.

An interesting observation—which was not in the aim of the study- that needs confirming, is the relationship between VOT derived parameters and patient outcome. In the previously mentioned study by Payen et al. [23], a relationship between the reperfusion slope and survival was found. Specifically, the reperfusion slope was significantly lower in nonsurvivors compared to survivors. Microcirculatory abnormalities have been associated with organ dysfunction and impaired outcome in cardiogenic as well as in septic shock [24, 25]. After major abdominal surgery Jhanji et al. reported that microvascular abnormalities were present in patients who subsequently developed postoperative complications, whereas microcirculation was intact in patients with an uneventful postoperative course [26]. It is interesting to note that in the aforementioned study global hemodynamic variables could not separate the two groups of patients.

There are two different mechanisms—although not, necessarily, mutually exclusive—which can affect microcirculation and tissue perfusion in these patients, leading to organ dysfunction and poor outcome.

The first is the postpump or postperfusion syndrome. During CPB the blood is brought into direct contact with a large artificial surface, pulsatile flow is converted to laminar flow, the heart is exposed to global cold ischemia with cardioplegic protection and the patient's body temperature is lowered by several degrees. In addition CPB causes endotoxemia, due to bacterial translocation from the gut. These features in combination with the surgical trauma contribute to an intense inflammatory reaction characterized by systemic endothelial and leukocyte interaction with widespread and local release of inflammatory mediators and activation of the complement system and the coagulation cascade. The microcirculation can be compromised due to microthrombi formation, neutrophil accumulation, swollen endothelial cells, as well as loss of the physiological vasodilation. The syndrome manifests clinically four to six hours postoperatively, with low peripheral resistance and hypotension, tachycardia and lactic acidosis, and results in tissue cell injury and MOD [3].

Peripheral blood flow and oxygen supply can also be affected postoperatively by a low cardiac output state. This can occur in patients with compromised systolic or diastolic ventricular function and also as a result of myocardial “stunning” due to ischemia-reperfusion injury of the heart. It is characterized by a CI less than 2.2 L/min/m^2^, cardiac filling pressures exceeding 20 mmHg, SVR exceeding 1500 dynes-s/cm^5^, and heart rate above 100/min. If left without intervention, it can also lead to tissue damage and organ failure [4].

In case of a low output state, the SvO_2_ decreases significantly, reflecting an elevated O_2_ER (up to 50–60% or more) and oxygen-supply dependency of tissue metabolism; patients also exhibit peripheral vasoconstriction with cool extremities and an amplified central to peripheral temperature difference [27], unlike our observations.

StO_2_ reflects the dynamic balance between the regional oxygen delivery and oxygen utilization. As the oxygen consumption rate increases gradually postoperatively, a similar/greater rise of the tissue oxygen flow should exist to allow for the stable/increased StO_2_. Thus, NIRS technology identifies tissue oxygenation status in its dynamic equilibrium and progression, which, the global oxygenation indices (DO_2_, VO_2_) estimated with the use of the Swan-Ganz catheter, fail to demonstrate.

All together, these data suggest that microvascular alterations in the postoperative period are associated with macrohemodynamics and may play a role in the development of postoperative organ dysfunction. Future studies are needed in cardiac surgery in order to further investigate the relationship between microcirculatory alterations postoperatively and patient outcome.

### 5.3. Limitations

A limitation of our study is the small number of patients included, as well as the lack of measurements at all time points for each patient. An additional limitation is that the statistical analysis included pooled data. A more extensive monitoring period, past the six hours, would have been useful in increasing our understanding of microcirculatory alterations and their trend after cardiac surgery with cardiopulmonary bypass. It would also have been useful to study a more homogeneous group of patients as the CABG patients are more likely to suffer from systemic cardiovascular disease as opposed to valve surgery patients.

## 6. Conclusion

The microcirculation, as assessed by near-infrared spectroscopy and the vascular occlusion technique, after cardiac surgery with cardiopulmonary bypass is related to macrohemodynamics and global indices of organ perfusion. Before incorporating microcirculatory parameters into clinical algorithms, a better understanding of the link between systemic hemodynamics and microvascular perfusion is needed, as well as a clarification of the relationship between microcirculatory alterations and organ failure in cardiac surgery.

## Figures and Tables

**Table 1:** Demographic data and cardiovascular risk factors. Data are presented as absolute numbers (percentage) or median (range), as appropriate.

Age	68 (28–82)
Gender	11 Male/12 Female

BMI (kg/m^2^)	27.1 (19–34.9)

Standard EuroSCORE	6 (1–12)

Logistic EuroSCORE (%)	5.4 (1.2–39.4)

Preoperative Risk Factors (No)	
Diabetes mellitus	3 (13)
Hypertension	13 (57)
Peripheral vascular disease	4 (17)
Dyslipidemia	7 (30)
Previous MI	4 (17)
Current smoking	6 (26)

EF (%)	60 (40–65)

Type of surgery (No)	
Coronary artery bypass grafting (CABG)	4
Aortic/Mitral valve replacement (AVR/MVR)	8
Atrial septum defect closure	1
CABG and AVR	2
Ascending aorta replacement	1
Ascending aorta replacement and MVR	1
Bentall procedure	4
MVR and tricuspid valve repair	2

CPB duration (min)	155 (60–226)

Aortic cross clamp time (min)	89 (32–156)

AV: aortic valve, BMI: body mass index, CABG: coronary artery bypass grafting, CPB: cardiopulmonary bypass, MI: myocardial infarction, MV: mitral valve, TV: tricuspid valve.

**Table:** (a)

	In cICU, after admission (H_0_), and every 2 hours up to 6 hours								
	H_0_		H_2_		H_4_		H_6_		*P*

Number of patients	23		21		18		17		
Thenar StO_2_ (%)	85 (71–98)		88 (78–98)		89 (76–98)		89 (79–98)*		<0.001
O_2_ consumption rate (%/min)	17 (5–44)		23 (4–50)*		25 (4–45)		28 (11–54)^∗§§††^		<0.001
Reperfusion rate (%/min)	203 (27–732)		350 (50–1215)**		467 (63–760)**		577 (123–1120)**		<0.01

	S	NS	S	NS	S	NS	S	NS	

Thenar StO_2_ (%)	85 (71–98)	88 (80–90)	88 (78–98)	90 (80–92)	89 (76–98)	91 (80–93)	89 (81–98)	92 (79–93)	ns
O_2_ consumption rate (%/min)	17 (5.1–44.2)	12.7 (8–24.9)	22.9 (3.8–50)	23.3 (9.4–26.8)	26.5 (4.2–44.8)	22 (12.5–30)	29.4 (11.2–53.9)	24 (11,3–38)	ns
Reperfusion rate (%/min)	236 (27–732)	148 (57–676)	362 (50–1215)	331 (73–457)	477 (63–760)	456 (168–589)	607 (123–1120)	312 (180–632)	ns

*P < 0.01 from H_0_, **P < 0.05 from H_0_, ^§^ *P* < 0.01 from H_2_, ^§§^ *P* < 0.05 from H_2_, ^†^ *P* < 0.01 from H_4_, ^††^ *P* < 0.05 from H_4_.

**Table:** (b)

	H_0_		H_2_		H_4_		H_6_		*P*
CO (L/min)	4.7 (2.4–11.4)		4.7 (2.3–10.5)		4.7 (3.3–9.2)		5.1 (2.4–11.6)		ns
CI (L/min/m^2^)	2.5 (1.5–5.7)		2.6 (1.2–5.3)		2.7 (2–4.7)		2.9 (1.6–5.8)		ns
PCWP (mmHg)	11 (4–27)		10.5 (5–22)		13 (4–20)		11 (3–21)		ns
CVP (mmHg)	9 (0–15)		8.5 (3–16)		10 (1–14)		11 (0–14)		ns
SVR (dynes-s/cm^5^)	1339 (89–2200)		1125 (594–1967)		1154 (558–1941)		1067 (564–1901)		ns
PVR (dynes-s/cm^5^)	214 (84–564)		206 (76–672)		182 (60–956)		220 (63–328)		ns
SvO_2_ (%)	71 (55–79)		71 (54–76)		69 (50–75)		71 (50–75)		ns
ScvO_2_ (%)	74 (65–85)		76 (59–87)		69 (49–78)		72 (48–85)		ns
Hb (g/dL)	10.9 (8.7–14.9)		10.7 (8.3–14.4)		10.7 (9–15.3)		10.7 (8.3–13.6)		ns
Lac (mg/dL)	2.2 (0.9–7)		3 (1.3–9.1)*		3.1 (0.7–9.9)*		3.6 (0.7–9.7)*		<0.05
MAP (mmHg)	79 (66–109)		73 (52–100)		76 (57–99)		77 (54–99)		ns
MPP (mmHg)	24 (13–33)		23 (14–35)		24 (17–61)		24 (15–36)		ns
Central temp (°C)	37.1 (35.6–38.8)		37.4 (36.2–39.1)*		37.6 (36.9–38.8)^∗§^		37.6 (37.2–38.4)^∗§^		<0.001
Periph temp (°C)	36.7 (35–38.7)		36.9 (35.3–38.5)**		37 (36–38.5)^∗§^		37.2 (36.2–38.4)^∗§^		<0.001
Central-Periph (°C)	0.4 (0–1.3)		0.4 (0–0.9)		0.4 (0–1.4)		0.4 (0–1)		ns
DO_2_ (mL/min/m^2^)	411 (193–721)		411 (137–737)		398 (278–660)		429 (264–750)		ns
VO_2_ (mL/min/m^2^)	114 (61–214)		110 (87–221)		116 (91–237)		129 (83–208)		ns
O_2_ ER (%)	27 (21–57)		29 (21–71)		29 (25–50)		28 (21–50)		ns

	S	NS	S	NS	S	NS	S	NS	ns

CO (L/min)	5 (3.1–11.4)	3.1 (2.4–4.7)	5.1 (3.3–10.5)	3.1 (2.3–3.9)	4.7 (3.5–9.2)	4.4 (3.3–4.7)	5.6 (4.3–11.6)	4.3 (2.4–4.4)	0.058
CI (L/min/m^2^)	2.5 (1.8–5.7)	1.8 (1.5–2.5)	2.8 (1.9–5.3)	1.8 (1.2–2.1)	2.7 (2–4.7)	2.3 (2.2–2.5)	3.1 (2.5–5.8)	2.3 (1.6–2.3)	0.064
PCWP (mmHg)	12 (4–27)	10 (8–10)	12 (8–22)	9 (5–10)	13 (4–20)	11 (8–11)	14 (3–21)	10 (9–11)	ns
CVP (mmHg)	9 (0–15)	8 (5–10)	9 (4–16)	8 (3–11)	10 (1–13)	9 (8–14)	11 (0–14)	8 (8–12)	ns
SVR (dynes-s/cm^5^)	1144 (89–2001)	1717 (1548–2200)	1113 (594–1506)	1504 (1128–1967)	1152 (558–1941)	1358 (1139–1525)	1006 (564–1327)	1580 (1325–1901)	0.074
PVR (dynes-s/cm^5^)	204 (84–479)	294 (103–564)	192 (76–301)	277 (167–672)	200 (60–956)	182 (170–286)	221 (63–296)	205 (163–328)	ns
SvO_2_ (%)	71 (55–79)	68 (42–72)	71 (54–76)	59 (29–73)	70 (50–75)	66 (63–74)	71 (50–75)	71 (66–77)	ns
ScvO_2_ (%)	74 (60–87)	70 (45–73)	75 (61–87)	59 (29–82)	71(49–82)	66 (60–69)	72 (48–85)	64(60–68)	0.035
Hb (g/dL)	11.3 (8.7–14.9)	10.9 (9.4–12.1)	10.9 (8.3–14.4)	10.2 (8.3–11.9)	11 (9–15.3)	9.9 (9.1–10.5)	10.7 (8.3–13.6)	11.9 (10.2–12.4)	ns
Lac (mg/dL)	1.9 (0.9–7)	2.9 (1.2–6.6)	2.6 (1.3–9.1)	5.5 (2.1–7.7)	3.2 (0.7–9.9)	3 (1.9–8.2)	3.7 (0.7–9.7)	2.1 (1.7–6.6)	0.076
MAP (mmHg)	81 (66–109)	74 (70–107)	75 (60–100)	66 (52–84)	75 (57–99)	76 (65–98)	76 (54–93)	79 (66–99)	ns
MPP (mmHg)	25 (13–33)	23 (14–29)	26 (20–35)	18 (14–25)	25 (17–61)	21 (20–21)	26 (15–36)	20 (19–21)	ns
Central temp (°C)	36.9 (35.7–38.8)	37.3 (35.6–37.7)	37.4 (36.2–37.1)	36.7 (36.5–38.1)	37.7 (37–38.8)	37 (36.9–37.1)	37.6 (37.2–38.4)	37.6 (37.5–37.6)	ns
Periph temp (°C)	36.7 (35.3–38.7)	36.9 (35.2–37.2)	37 (35.9–38.5)	36.4 (36.1–37.2)	37.2 (36.2–38.5)	36.7 (36.6–36.8)	37.2 (36.4–38.4)	37.2 (37.1–37.2)	ns
Central-Periph (°C)	0.2 (0–1.3)	0.45 (0.4–0.5)	0.5 (0–0.8)	0.4 (0.3–0.9)	0.5 (0–1.4)	0.3 (0.2–0.3)	0.4 (0–1)	0.5 (0.4–0.5)	ns
DO_2_ (mL/min/m^2^)	457 (292–721)	255 (193–416)	417 (281–737)	252 (137–340)	421 (335–660)	316 (278–361)	431 (350–750)	356 (323–389)	ns
VO_2_ (mL/min/m^2^)	118 (76–214)	107 (61–113)	118 (94–221)	96 (87–109)	117 (99–238)	93 (91–117)	136 (97–208)	96 (83–109)	ns
O_2_ ER (%)	27 (21–43)	31 (27–57)	29 (21–45)	40 (25–70)	29 (25–50)	34 (25–37)	28 (24–50)	28 (21–34)	ns

CI: cardiac index, CO: cardiac output, CVP: central venous pressure, DO_2_: whole-body oxygen delivery, Hb: hemoglobin, Lac: lactate, MAP: mean arterial pressure, MPP: mean pulmonary pressure, O_2_ER: oxygen extraction ratio, PCWP: pulmonary capillary wedge pressure, PVR: pulmonary vascular resistance, ScvO_2_: central venous oxygen saturation, SvO_2_: mixed venous oxygen saturation, SVR: systematic vascular resistance, temp: temperature, VO_2_: whole-body oxygen consumption. *P < 0.01 from H_0_, **P < 0.05 from H_0_, ^§^ *P* < 0.01 from H_2_, ^§§^ *P* < 0.05 from H_2_, ^†^ *P* < 0.01 from H_4_, ^††^ *P* < 0.05 from H_4_.

## Abbreviations

AV: Aortic valve
BMI: Body mass index
CABG: Coronary artery bypass grafting
CI: Cardiac index
cICU: Cardiac ICU
CO: Cardiac output
CPB: Cardiopulmonary bypass
CVP: Central venous pressure
DO_2_: Total oxygen delivery
Hb: Hemoglobin
Lac: Lactate
MAP: Mean arterial pressure
MI: Myocardial infarction
MPP: Mean pulmonary pressure
MV: Mitral Valve
NIRS: Near-infrared spectroscopy
O_2_ ER: Oxygen extraction ratio
PCWP: Pulmonary capillary wedge pressure
PVR: Pulmonary vascular resistance
ScvO_2_: Central venous oxygen saturation
SIRS: Systematic inflammatory response syndrome
StO_2:_: Tissue oxygen saturation
SvO_2:_: Mixed venous oxygen saturation
SVR: Systematic vascular resistance
Temp: Temperature
TV: Tricuspid valve
VO_2:_: Total oxygen consumption
VOT: Vascular occlusion technique.
